# Estimating Diarrheal Illness and Deaths Attributable to *Shigellae* and Enterotoxigenic *Escherichia coli* among Older Children, Adolescents, and Adults in South Asia and Africa

**DOI:** 10.1371/journal.pntd.0002705

**Published:** 2014-02-13

**Authors:** Laura M. Lamberti, A. Louis Bourgeois, Christa L. Fischer Walker, Robert E. Black, David Sack

**Affiliations:** 1 Johns Hopkins Bloomberg School of Public Health, Department of International Health, Baltimore, Maryland, United States of America; 2 PATH, Washington, D.C., United States of America; University of California San Diego School of Medicine, United States of America

## Abstract

**Introduction:**

While *Shigellae* and strains of enterotoxigenic *Escherichia coli* (ETEC) are important causes of diarrhea-associated morbidity and mortality among infants and young children (<5 years of age), their health impact in older age groups is unclear. We sought to quantify the overall burden of shigellosis and ETEC diarrhea among older children, adolescents, and adults in Africa and South Asia, the two regions with the highest levels of diarrhea-related morbidity and mortality worldwide.

**Methods:**

We employed two distinct methodological approaches to estimate the burden of diarrhea due to *Shigellae* and ETEC among persons ≥5 years of age in the WHO regions of South Asia (SEAR) and Africa (AFR). Under method 1, we conducted a systematic review to identify the median proportion of total deaths due to diarrhea and then applied this figure to the number of all-cause deaths that occurred in 2010 among this age group. To estimate the total number of diarrhea deaths attributable to *Shigellae* and ETEC, we subsequently applied previously published estimates of the median percentage of diarrhea hospitalizations due to *Shigellae* and ETEC to the estimated number of diarrhea deaths. For method 2, we applied previously published incidence rates to 2010 population figures and estimated the total number of episodes due to *Shigellae* and ETEC using published estimates of the average proportion of pathogen-positive outpatients from studies of >4 pathogens. We then estimated the number of pathogen-specific deaths by determining the number of hospitalized patients and applying the case-fatality rate.

**Results:**

By method 1, there were 19,451 deaths due to *Shigellae* and 42,973 due to ETEC in AFR, and 20,691 due to S*higellae* and 45,713 due to ETEC in SEAR in 2010. By method 2, there were 15.0 million ETEC episodes and 30.4 million episodes due to *Shigellae* in AFR, and 28.7 million episodes due to ETEC and 58.1 million episodes due to *Shigellae* in SEAR in 2010. We were unable to identify published case-fatality rates for ETEC and thus could only estimate *Shigellae-related* deaths using method 2, by which there were 5,308 and 10,158 *Shigellae-related* deaths in AFR and SEAR in 2010, respectively.

**Discussion:**

Methods 1 and 2 underscore the importance of *Shigellae* and ETEC as major causes of morbidity and mortality among older children, adolescents, and adults in AFR and SEAR. Understanding the epidemiology of these pathogens is imperative for the development and use of future vaccines and other preventative interventions.

## Author Summary

We aimed to estimate the burden of diarrhea due to *Shigellae* and ETEC among persons ≥5 years of age in Africa and South Asia using two methodological approaches. Method 1 employed diarrhea proportionate mortality and published estimates of pathogen-specific hospitalization. Method 2 utilized published incidence rates and case-fatality rates. Method 1 resulted in 19,451 deaths due to *Shigellae* and 42,973 deaths due to ETEC in AFR, and 20,691 deaths due to *Shigellae* and 45,713 deaths due to ETEC in SEAR in 2010. Method 2 resulted in 15.0 million episodes due to ETEC and 30.4 million *episodes* due to *Shigellae* in AFR, and 28.7 million episodes due to ETEC and 58.1 million episodes due to *Shigellae* in SEAR in 2010. These figures highlight the importance of *Shigellae* and ETEC as major causes of morbidity and mortality among older children, adolescents and adults in Africa and South Asia.

## Introduction

Although frequently underappreciated as a significant health problem, recent publications have highlighted the potential importance of diarrhea as a continuing cause of morbidity and mortality among older children, adolescents and adults living in low- and middle-income countries [1], [2]. Similar to children under five years of age, diarrhea incidence and mortality estimates among older age groups are highest in Africa and South Asia [1], [2]. The burden of diarrheal episodes beyond early childhood may have substantial public health and economic impact at the family, community and national levels that extends well beyond mortality alone.

Recent global and regional assessments have served to reiterate the importance of cholera, shigellosis and enterotoxigenic *Escherichia coli* (ETEC) as causes of diarrhea morbidity and mortality among infants and young children <5 years of age [3]–[5]. While, such reviews have illustrated that cholera is of comparatively greater importance among children under-five than among older children and adults, limited data are available to describe the potential contribution of *Shigellae* and ETEC to diarrhea incidence and mortality among older children, adolescents and adults [6]. Improved estimates of the diarrhea burden due to *Shigellae* and ETEC are needed in this under-evaluated age group and, in particular, for the regions of South Asia and Africa, which represent 30% of annual diarrhea episodes among persons ≥5 years of age and 82% of global diarrhea deaths among children under-five [2], [7]. Assessing the burden from *Shigellae* and ETEC is needed because both pathogens represent longstanding World Health Organization (WHO) targets for vaccine development. Given the advent of increasing donor support for enteric vaccine research, vaccine development efforts for both pathogens have been reenergized, and there is a growing need to better define the full range of risk-groups in which new vaccines or other preventive interventions could be used [8].

The purpose of this review is to quantify the overall burden of diarrhea due to *Shigellae* and ETEC among older children, adolescents, and adults in Africa and South Asia. Improved understanding of the etiology of diarrhea in this population is of critical importance for vaccine development and implementation of more targeted prevention and control strategies in the two regions with the highest level of diarrhea morbidity and mortality in the world.

## Methods

With the goal of estimating the annual number of cases, hospitalizations, and deaths due to *Shigellae* and ETEC among older children and adults in South Asia and Africa, we employed two separate methodological approaches that each relied on published epidemiological data.

### Method 1

We estimated the proportion of deaths due to diarrhea among older children and adults and then estimated the proportion of these deaths which were due to infections with *Shigellae* or ETEC. To do this, we first conducted a systematic literature review of studies published from January 1, 2009–December 31, 2012 in order to update a previously published review of mortality studies published from 1980–2008 [2]. We searched PubMed/Medline, Embase, CAB abstracts, System for Information on Grey Literature in Europe (SIGLE) and the World Health Organization (WHO) Regional databases using combinations of the following MeSH terms: diarrhea, gastroenteritis and mortality. We also searched the internet for published reports on demographic surveillance sites (DSS).

We performed a double review of the search results and double data abstraction of included papers. We examined titles and abstracts for relevancy and reviewed pertinent articles for inclusion and exclusion criteria. In accordance with the previous published review [2], we included reports of diarrhea-specific mortality outcomes among older children (≥5 years of age) and adults published in any language during the specified time frame. We accepted all definitions of diarrhea because a single standard definition of diarrhea among older children and adults does not exist. To prevent seasonality bias, we narrowed our results to reports containing at least 12 months of mortality recall. We excluded studies reporting less than 20 total deaths and studies conducted in special populations, such as travelers, cancer patients, HIV-infected individuals and refugees. We also excluded studies focused on antibiotic-associated diarrhea, hospital-acquired diarrhea, outbreaks and specific etiologic agents as opposed to all-cause diarrhea. Review papers and individual case reports were also excluded.

To estimate the total number of overall deaths among persons ≥5 years of age in SEAR and AFR in 2010, we divided the region-specific deaths reported by the United Nations Department of Economic and Social Affairs Population Division for the period of July 2005–June 2010 by five [9]. We used STATA 12 to calculate the region-specific median proportion of deaths attributed to diarrhea across all studies identified for persons 5–14 and ≥15 years of age [10]. For each region, we approximated the total number of diarrhea deaths by applying the region- and age-specific median proportion of diarrhea deaths to the estimated number of deaths in 2010. We used the proportion of diarrhea patients hospitalized for diarrhea who tested positive for *Shigellae* or ETEC as reported by a review of diarrhea etiology among older children and adults [6]. We applied these proportions to total diarrhea deaths to approximate the total number of diarrhea deaths attributable to *Shigellae* and ETEC.

### Method 2

We estimated the total number of diarrhea episodes due to *Shigellae* and ETEC that occurred among persons ≥5 years of age in SEAR and AFR in 2010 and applied case-fatality rates to determine the total number of pathogen-specific deaths. In order to achieve this, we applied previously reported diarrhea incidence rates to 2010 regional populations in three age groups (5–14 years; 15–54 years; and ≥55 years) to estimate the annual number of diarrhea episodes among persons ≥5 years of age [6], [9]. Age-specific diarrhea incidence rates for these two regions were estimated based on a review of community-based studies among older children and adults [6]. In order to estimate the annual number of episodes due to *Shigellae* and ETEC, we applied the mean percentage of *Shigellae*-positive and ETEC-positive outpatients to the estimated number of diarrhea episodes in SEAR and AFR. The mean percentages of *Shigellae*- and ETEC-positive outpatients were derived from a review that aimed to isolate at least 4 pathogens from stools collected in a hospital outpatient setting [6], since studies focused on a wide range of pathogens are more reflective of true causative burden than studies of one specific pathogen that are typically located in regions where the pathogen of interest is endemic.

To identify published estimates of *Shigellae* and ETEC case-fatality rates (CFRs) among older children and adults, we conducted a literature search of peer-reviewed studies and academic texts. We were unable to identify a relevant CFR for ETEC and were thus unable to estimate the total number of ETEC-attributable deaths by Method 2. We identified a published CFR for hospitalized cases due to *Shigellae* among persons ≥5 years of age [11]. Before applying this CFR to our data, we approximated the total number of hospitalized shigellosis cases by multiplying the estimated number of episodes due to *Shigellae* in each region by the proportion of annual episodes due to *Shigellae* at a treatment facility (i.e. 0.053) [11]. We then applied the CFR for hospitalized cases due to *Shigellae* to the total number of hospitalized cases and generated an estimate of *Shigellae*-attributable deaths among persons ≥5 years of age.

## Results

### Method 1

**Figure 1:**
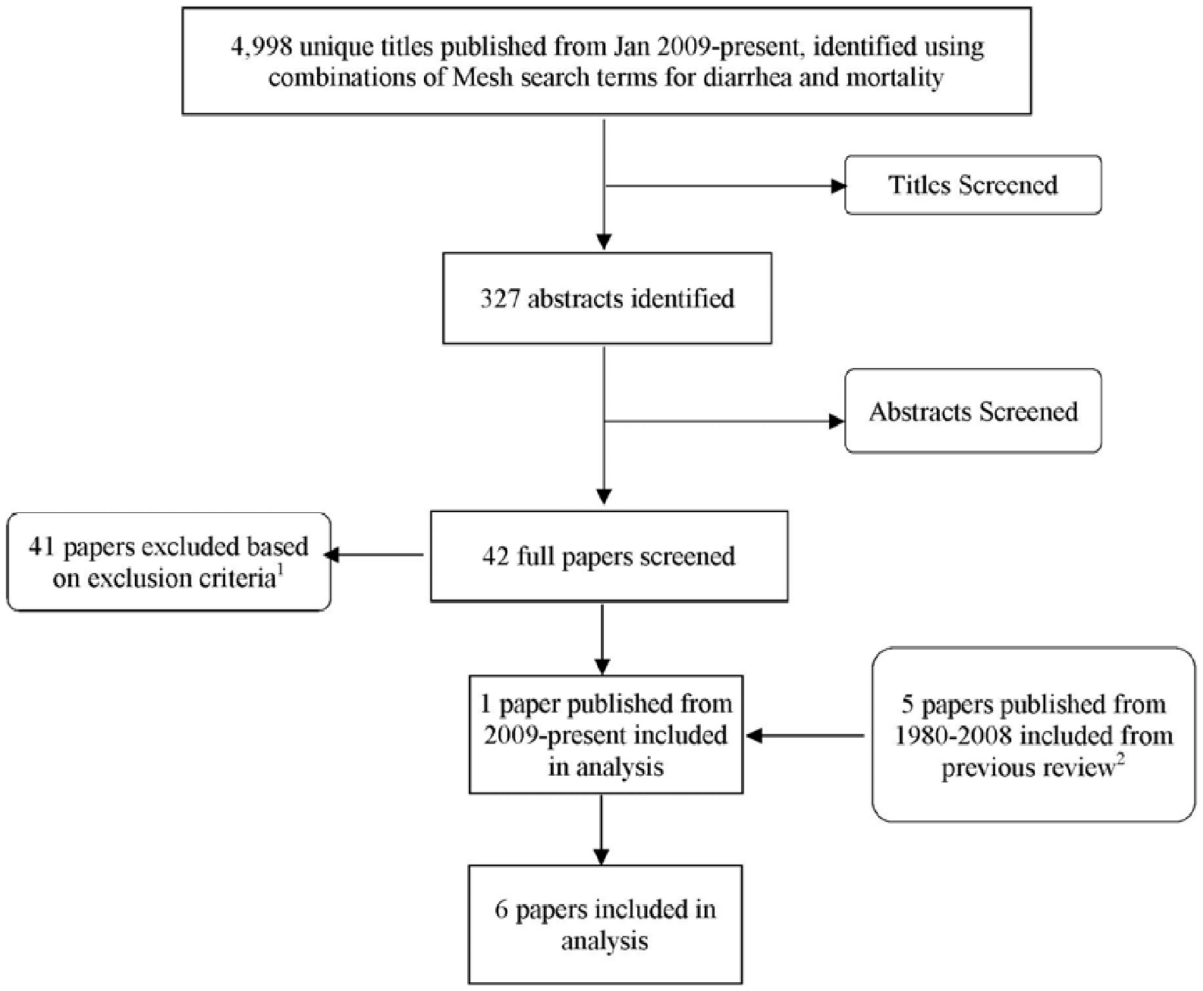
Results of the systematic review. Footnotes: ^1^Main reasons for exclusion: no outcome of interest (n = 23); review paper (n = 5); under-five data only (n = 5); data combined across under-five and older age groups (n = 4); retrospective review of hospital records (n = 3); special population (n = 1). ^2^ Source: Fischer Walker CL, Black RE (2010) Diarrhoea morbidity and mortality in older children, adolescents, and adults. Epidemiol Infect 138: 1215–1226.

The database search yielded 5,000 unique titles. Title and abstract review narrowed the results to 327 and 42 papers, respectively. Following full-paper review, one additional article met all inclusion/exclusion criteria (Figure 1) [12]. No published reports of DSS data were identified that met all inclusion and exclusion criteria.

Diarrhea accounted for 12.5% of deaths in AFR and 19.85% of deaths in SEAR among persons 5–14 years of age. Among persons ≥15 years of age, diarrhea-proportionate mortality was 6.35% in AFR and 3.7% in SEAR. In 2010, there were approximately 452,348 diarrhea deaths in AFR and 481,189 diarrhea deaths in SEAR. Using previously reported estimates of the proportion of diarrhea hospitalizations due to *Shigellae* and ETEC as a proxy for death (i.e. 4.3% and 9.5%, respectively) [6], we estimated 19,451 deaths due to *Shigellae* and 42,973 deaths due to ETEC in AFR; and 20,691 deaths due to *Shigellae* and 45,713 deaths due to ETEC in SEAR in 2010 (Table 1).

**Table 1:** Estimated number of deaths due to *Shigellae* and ETEC among older children and adults in Africa and South Asia using Method 1.

Author	Country	Age group	% deaths attributable to diarrhea	Median Age-Specific % Deaths Attributable to Diarrhea	Age-specific total # deaths per year∧∧	Total # diarrhea deaths*	Total # of Diarrhea Deaths attributable to *Shigellae* **	Total # of Diarrhea Deaths attributable to ETEC***
**AFR**								
Etard∧	Senegal	5–14	12.5	12.5	777,983	97,248	4,182	9,239
CDC∧	Tanzania	≥15	4.6	6.35	5,592,124	355,100	15,269	33,734
Kelly∧	Zambia		20					
Adjuik∧	South Africa		1.2					
Adjuik∧	Senegal		8.1					
**TOTAL**					6,370,107	452,348	19,451	42,973
**SEAR**								
Anwar∧	Indonesia	5–14	22.2	19.85	382,062	75,839	3,261	7,205
Morris [12]	India		17.5					
Adjuik∧	Bangladesh	≥15	3.7	3.7	10,955,403	405,350	17,430	38,508
**TOTAL**					11,337,465	481,189	20,691	45,713

∧ Papers identified by previously published systematic review [2].

∧∧ Number of deaths per year among persons ≥5 yrs of age in countries identified by WHO regions AFR and SEAR; calculated by dividing 2005–2010 deaths by 5. Source: UN Population Division [9].

* Calculated by multiplying the age-specific median % deaths attributable to diarrhea by the age-specific total # deaths per year.

** Calculated by multiplying median % of diarrhea hospitalizations due to *Shigellae* (i.e. 4.3%) by total # of diarrhea deaths in each region [6].

*** Calculated by multiplying median % of diarrhea hospitalizations due to ETEC (i.e. 9.5%) by total # of diarrhea deaths in each region [6].

### Method 2

We identified a review of studies aiming to isolate at least 4 pathogens, which estimated the mean percentage of ETEC-positive outpatients at 4.6% and the mean percentage of *Shigellae*-positive outpatients at 9.3% [6]. Using these figures, we estimated an annual incidence of 15.0 million ETEC episodes in AFR, 28.7 million ETEC episodes in SEAR, 30.4 million episodes due to *Shigellae* in AFR, and 58.1 million episodes due to *Shigellae* in SEAR among persons ≥5 years of age (Table 2).

**Table 2:** Estimated number of episodes due to hospitalizations, and deaths due to *Shigellae* among older children and adults in Africa and South Asia using Method 2.

	5–14 yr	15–54 yr	≥55 yr	Annual # Diarrhea Episodes	Annual # ETEC Episodes*	Annual # *Shigellae* Episodes*	Annual # *Shigellae Hospitalizations* **	Annual # *Shigellae* Deaths***
**AFR** ∧								
Median (IQR) Diarrhea Incidence Rate per 100 p-yr [6]	67.5 (15.4–91.7)	29.9 (7.6–47.7)	30.1 (7.6–59.9)					
2010 Population∧∧	238,750,338	479,518,541	72,516,977					
Annual # Diarrhea Episodes	161,156,478	143,376,044	21,827,610	326,360,132	15,012,566	30,351,492	1,608,629	5,308
**SEAR**								
Median (IQR) Diarrhea Incidence Rate per 100 p-yr [6]	67.5 (15.4–91.7)	29.9 (7.6–47.7)	30.1 (7.6–59.9)					
2010 Population∧∧	354,919,237	1,062,050,879	223,776,679					
Annual # Diarrhea Episodes	239,570,485	317,553,213	67,356,780	624,480,478	28,726,102	58,076,684	3,078,064	10,158

∧ Median incidence data not available; review used SEAR incidence rates applied to AFR population to get total # annual episodes [6].

∧∧ 2010 population among persons ≥5 yrs of age. Source: UN Population Division [9].

* Calculated using weighted mean % positive from outpatient studies reporting >4 pathogens (i.e. ETEC: 4.6%; *Shigellae*: 9.3%) [6].

** Calculated using proportion of annual cases due to *Shigellae* reported at a treatment facility (i.e. 0.053) [11].

*** Calculated by applying case fatality rate for hospitalized cases due to *Shigellae* (i.e. 0.33%) [11].

We were unable to identify a published CFR for ETEC diarrhea among older children and adults and, therefore, did not generate an estimate of ETEC deaths in this population using method 2. The CFR for ETEC diarrhea is extremely low among treated cases, and although most cases go untreated, the CFR for untreated cases is unknown [13].

We applied the CFR for hospitalized cases due to *Shigellae* ≥5 years of age, 0.33%, to the number of hospitalized cases due to *Shigellae* and estimated 5,308 *Shigellae-associated* deaths in AFR and 10,158 *Shigellae-associated* deaths in SEAR (Table 2) [11]. This estimate is an upper bound, since the CFR was reported as a range from 0–0.33% [11].

## Discussion

We employed two distinct methodological approaches to approximate the burden of shigellosis among older children, adolescents and adults. Estimates of deaths due to *Shigellae* occurring among this population in 2010 were elevated by method 1 compared to method 2 (*Shigellae-associated* deaths by method 1: 19,451 in AFR and 20,691 in SEAR in 2010; *Shigellae-associated* deaths by method 2: 5,308 in AFR and 10,158 in SEAR). We were unable to utilize method 2 to derive estimates of the burden of ETEC diarrhea among older children, adolescents and adults; however, method 1 yielded approximately 42,973 ETEC associated deaths in AFR and 45,713 ETEC associated deaths in SEAR in 2010.

Although approaches such as these used to estimate diarrhea mortality may be prone to limitations and bias, such techniques are necessary due to the lack of reliable cause-of-death data in resource-poor settings where vital registration is suboptimal or nonexistent. Verbal autopsy data are widely used to estimate cause of death among children under five years of age [7], but the volume of data available is many times what we found here for older children and adults. The scarcity of these data limits what can be soundly estimated with complex multivariate models.

In this paper, we focused specifically on estimating *Shigellae*- and ETEC-specific morbidity and mortality among older children, adolescent and adults. The Global Burden of Disease Study (GBD) published estimates of older child and adult mortality attributable to *Shigellae* and ETEC but, because the study did not outline the specific components of the model used to approximate pathogen-specific incidence and mortality [3], we were unable to draw comparisons between the GBD methods and our own. Globally, the GBD Study reported 779,800 total diarrhea deaths, 79,200 deaths due to *Shigellae* deaths and 82,100 deaths due to ETEC deaths among persons ≥5 years of age in 2010 [3]. In comparison, our method 1 estimates for AFR and SEAR are greater for total diarrhea and ETEC-associated deaths (i.e. 933,537 and 88,686, respectively) (Table 1). Our method 1 and method 2 estimates of deaths due to *Shigellae* for these regions are less than the GBD global estimates (i.e. 40,142 by method 1and 15,466 by method 2) (Table 1).

Two recent studies have published data on diarrhea etiology among children <5 years of age. Lanata, *et. al.* published a review of diarrhea etiology among children hospitalized for diarrhea and estimated that together *Shigellae* and ETEC may account for 9.8% of child diarrhea deaths [14]. The Global Enteric Multicenter Study (GEMS) published estimates of diarrhea due to *Shigellae* and ETEC illustrating that both pathogens accounted for about 22% of diarrhea deaths among cases of moderate-to-severe diarrhea aged 0–23 months [4]. Our analysis bolsters and expands upon the findings of these studies, which were conducted among children under-five years of age, to provide evidence that *Shigellae* and ETEC continue to impact health from early childhood through school, adolescence and adulthood.

Both methodological approaches proposed by our review have strengths and limitations. Method 1 estimates were more than three-fold higher than method 2 estimates for AFR and about two-fold higher for SEAR for *Shigellae*. The use of hospitalizations as a proxy for estimating pathogen-specific deaths may have led method 1 to overestimate the true number of *Shigellae-associated* deaths in regions where hospitalizations for moderate episodes due to *Shigellae* that do not progress to death are higher than expected. While this approach is less than ideal, it is the only alternative in the absence of relevant data on pathogen-specific deaths and is similar to the method used for estimating deaths attributable to rotavirus [15].

On the other hand, the median incidence rates used by method 2 were derived from a systematic review that excluded outbreak studies and studies conducted in high diarrhea season, resulting in presumably underestimated incidence figures [2]. In addition, method 2 utilizes the proportion of annual cases due to *Shigellae* that occurred at a treatment facility (i.e. 0.053) as a proxy for episodes severe enough to progress to death [11]; since this estimate was derived from a systematic review of 9 studies conducted in 6 Asian countries, it is possible that facility treatment was not sought for a proportion of severe episodes due to *Shigellae*. Thus, method 1 and method 2 estimates may respectively represent the upper and lower bounds for *Shigellae*-associated deaths among persons ≥5 years of age in 2010.

A major strength of method 1 is that it enables approximation of regional pathogen-specific mortality in the absence of data on etiologic cause of death. As previously noted, the use of hospitalizations as a proxy for mortality may overestimate the true number of pathogen-specific deaths and is therefore a limitation of method 1; this assumes that the risk of requiring hospitalization for diarrhea due to *Shigellae* or ETEC is equivalent to the overall risk of death due to these pathogens. The results of method 1 are also limited by the restricted geographic scope of the region-specific proportionate mortality estimates, which were pooled from six studies conducted in four AFR and three SEAR countries. In addition, method 1 used pathogen-specific hospitalization proportions from a previously published review with its own limitations, including scarcity of data, heterogeneity of laboratory methods over time, and limited geographic scope [6].

Method 2 is limited by the dearth of literature on CFRs for shigellosis and ETEC diarrhea among older children, adolescents and adults. For diarrhea due to *Shigellae*, we were unable to identify CFRs for non-hospitalized cases among persons ≥5 years of age and were thus required to approximate the number of hospitalized cases prior to applying the case-fatality rate. We were unable to identify any CFRs for ETEC diarrhea. Estimates generated by this method would therefore be improved by additional research into the case fatality associated with community cases due to *Shigellae* and ETEC.

The data are more robust for deaths due to *Shigellae* and ETEC in SEAR as opposed to AFR and, therefore, estimates for SEAR may be more accurate than those for AFR. The majority of studies contributing to the pathogen-specific hospitalization proportions used by method 1 were conducted in SEAR (5 of 8 *Shigella* studies and 3 of 4 ETEC studies) and none were conducted in AFR. For method 2, median incidence data were lacking for AFR, and to compensate for this deficit, we applied SEAR incidence rates to the AFR 2010 population as was previously documented in the literature [2].

Although methods 1 and 2 are restricted by the availability of relevant data, we feel that the data are sufficiently robust to draw estimates for AFR and SEAR for both pathogens by method 1 and for *Shigellae* by method 2. We are unable to generate confidence intervals to illustrate the precision around our estimates due to the unavailability of uncertainty ranges for our models' inputs, and we acknowledge that confidence around our estimates is likely wide. However, we also assert that we have undertaken a transparent approach in forming these estimates and that this is an important strength of this analysis.

In comparison with selected enteric infections, such as typhoid fever and cholera, this study highlights that *Shigellae* and ETEC are also of importance to diarrhea morbidity among older children, adolescents and adults. Applying published incidence rates for cholera and typhoid to the 2010 population, it is evident that in 2010 the rank of these pathogens from highest to lowest incidence in both SEAR and AFR was: *Shigellae*, ETEC, typhoid (SEAR: 3.1 million; AFR: 2.9 million) and cholera (SEAR: 1.6 million; AFR: 1.2 million) (Table 2) [5], [16].

While our mortality estimates are grouped together for persons 5–14 and ≥15 years of age, it is noteworthy that there is an age curve of diarrhea deaths among older children, adolescents and adults. DSS data from Bangladesh illustrate that 3%, 11% and 86% of total diarrhea deaths occur among persons 5–14 years, 15–54 years and ≥55 years of age, respectively [17]. Additional data with regard to pathogen-specific mortality rates would be useful to help us understand if this curve is consistent across all pathogens. Furthermore, additional research is warranted to assess age-specific and etiology-specific incidence rates.

The estimates generated by this review shed light on the epidemiology of shigellosis and ETEC diarrhea among older children, adolescents, and adults in AFR and SEAR. Understanding the burden of these pathogens in two regions of high diarrhea incidence is of critical importance for improving prevention and treatment. Together, methods 1 and 2 suggest that *Shigellae* and ETEC are responsible for a significant degree of morbidity and mortality among persons ≥5 years of age in AFR and SEAR. As such, *Shigellae* and ETEC contribute to lost productivity and school absenteeism, especially among older children 5–14 years of age. The development of vaccines and other interventions aimed at the prevention of these pathogens among older children, adolescents and adults is thus essential to furthering social and economic development.
